# Human endometrium-derived stem cell improves cardiac function after myocardial ischemic injury by enhancing angiogenesis and myocardial metabolism

**DOI:** 10.1186/s13287-021-02423-5

**Published:** 2021-06-10

**Authors:** Xuemei Fan, Sheng He, Huifang Song, Wenjuan Yin, Jie Zhang, Zexu Peng, Kun Yang, Xiaoyan Zhai, Lingxia Zhao, Hui Gong, Yi Ping, Xiangying Jiao, Sanyuan Zhang, Changping Yan, Hongliang Wang, Ren-Ke Li, Jun Xie

**Affiliations:** 1 The Laboratory of Stem Cell Regenerative Medicine Research, Shanxi Key Laboratory of Birth Defect and Cell Regeneration, Key Laboratory of Cell Physiology of Ministry of Education, Shanxi Medical University, Taiyuan, China; 2 Shanxi Bethune Hospital, Shanxi Academy of Medical Sciences, The Third Hospital of Shanxi Medical University, Taiyuan, China; 3 The First Hospital of Shanxi Medical University, Taiyuan, China; 4 The Second Hospital of Shanxi Medical University, Taiyuan, China; 5 Key Laboratory of Molecular Imaging, Molecular Imaging Precision Medicine Collaborative Innovation Center, Shanxi Medical University, Taiyuan, China; 6 Toronto General Hospital Research Institute, University Health Network, Toronto, Canada

**Keywords:** Human endometrium-derived stem cells, Myocardial ischemic injury, Human bone marrow mesenchymal stem cells, Angiogenesis, Cardiac repair

## Abstract

**Background:**

The human endometrium in premenopausal women is an active site of physiological angiogenesis, with regenerative cells present, suggesting that the endometrium contains adult angiogenic stem cells. In the context of cardiac repair after ischemic injury, angiogenesis is a crucial process to rescue cardiomyocytes. We therefore investigated whether human endometrium-derived stem cells (hEMSCs) can be used for cardiac repair after ischemic injury and their possible underlying mechanisms.

**Methods:**

Comparisons were made between hEMSCs successfully isolated from 22 premenopausal women and human bone marrow mesenchymal stem cells (hBMSCs) derived from 25 age-matched patients. Cell proliferation, migration, differentiation, and angiogenesis were evaluated through in vitro experiments, while the ability of hEMSCs to restore cardiac function was examined by in vivo cell transplantation into the infarcted nude rat hearts.

**Results:**

In vitro data showed that hEMSCs had greater proliferative and migratory capacities, whereas hBMSCs had better adipogenic differentiation ability. Human umbilical cord vein endothelial cells, treated with conditioned medium from hEMSCs, had significantly higher tube formation than that from hBMSCs or control medium, indicating greater angiogenic potentials for hEMSCs. In vivo, hEMSC transplantation preserved cardiac function, decreased infarct size, and improved tissue repair post-injury. Cardiac metabolism, assessed by ^18^F-FDG uptake, showed that ^18^F-FDG uptake at the infarction area was significantly higher in both hBMSC and hEMSC groups, compared to the PBS control group, with hEMSCs having the highest uptake, suggesting hEMSC treatment improves cardiomyocyte metabolism and survival after injury. Mechanistic assessment of the angiogenic potential for hEMSCS revealed that angiogenesis-related factors angiopoietin 2, Fms-like tyrosine kinase 1, and FGF9 were significantly upregulated in hEMSC-implanted infarcted hearts, compared to the PBS control group.

**Conclusion:**

hEMSCs, compared to hBMSCs, have greater capacity to induce angiogenesis, and improved cardiac function after ischemic injury.

**Supplementary Information:**

The online version contains supplementary material available at 10.1186/s13287-021-02423-5.

## Background

Ischemic cardiomyopathy is a leading cause of morbidity and mortality worldwide [1]. Current therapies, including medication, percutaneous coronary intervention (stents and balloon dilatation), and bypass surgery, which all aim to restore blood supply into the ischemic myocardium, are effective in most patients. However, despite great improvements, it is inevitable that myocardial infarction (MI) leads to permanent loss of cardiac tissue and ultimately heart failure [2]. In recent decades, cell-based therapy has emerged as a new approach for repairing heart tissue. Among many candidates, human bone marrow mesenchymal stem cells (hBMSCs) represent the most commonly-used cell type for clinical trials. However, the beneficial effects of autologous hBMSC therapy have been shown to be marginal [3, 4], most likely due to the inability of aged hBMSCs to induce blood vessel formation and tissue repair. The search for ideal candidate cells for cardiac repair remains an active area of investigation. An ideal cell candidate should have the prerequisite characteristics of strong proliferation and paracrine capacity, immuno-privilege, and especially strong angiogenic potential, which could possibly be found in endometrium-derived stem cells.

It has been observed that the incidence of cardiovascular events in premenopausal women is significantly lower than in age-matched men. The disappearance of this disparity in women with natural and surgical menopause is worthy of interest [5, 6]. More intriguingly, current studies solely using hormone therapy have not significantly reduced the risk of cardiovascular events in postmenopausal women [7–9]. Our previous pre-clinical study, where uteri were implanted into adult rats, demonstrated the presence of cross-talk between uterus and heart post-myocardial ischemic injury [10]. These results suggest that a functional uterus may be an independent factor for reducing the risk of myocardial ischemic injury. The endometrium is an active site of physiological angiogenesis in premenopausal women, where uterine stem/progenitor cells periodically divide and differentiate to generate decidual tissue, without scar formation. All these evidences point out the possibility of using endometrium-derived stem/progenitor cells, possessing strong capabilities to promote angiogenesis and regeneration, as an attractive candidate for cardiac repair.

Our group has previously demonstrated that uterine cells implanted into injured myocardium increased blood vessel density at the implanted area, reduced scar size, and improved cardiac function, relative to smooth muscle cells and media alone [11]. Through uterine transplantation or intravenous uterine cell injection, the uterine cells were able to home into the injured myocardium, stimulate angiogenesis and improve cardiac function [10, 12]. In a mouse model, we demonstrated that intra-myocardial injection of allogeneic MHC I (neg) cells after MI restored cardiac function, with limited recruitment of CD4 and CD8 cells. This new source of immuno-privileged cells can induce neovascularization, thereby providing a possible allogeneic cell therapy approach in regenerative medicine [12]. Additionally, studies on human endometrial stem cells (hEMSCs) have shown that they conferred superior cardio-protection compared to hBMSCs, probably through the miR-21 mediated PTEN/Akt pathway [13]. In this study, we further investigated the effects of hEMSCs on altering cardiac metabolism and function within ischemic tissue. We isolated hEMSCs and evaluated their in vitro and in vivo angiogenic capacities, as well as their efficacy in perfusion restoration of ischemic tissue in adult rat hearts. Compared to age-matched hBMSCs, we demonstrated that hEMSCs have the greater angiogenic capacity to restore tissue reperfusion, suggesting them being an excellent cell source for ischemic heart disease.

## Materials and methods

### Human tissue collection

Human endometrium was obtained from 22 premenopausal women (mean age 41.8 ± 5.5 [29–49] years), who underwent hysterectomy for fibroids or adenomyosis in the hospitals. Samples were taken from patients without administration of exogenous hormones for 3 months prior to surgery. The specimens, including full endometrium thickness, were collected into Dulbecco’s modified Eagle’s medium/Ham’s Nutrient Mixture F-12 (DMEM/F12 1:1, Gibco, Cat#: 12400024), supplemented with 100 U/ml penicillin, 100 μg/mL streptomycin, and 10% fetal bovine serum (FBS), and was followed by processing.

Human bone marrow was harvested from 25 women (mean age 41.7 ± 5.3 [32–49] years). Samples from subjects with no genetic disease or malignancies, based on primary diagnosis, were used. One milliliters of human bone marrow was harvested from the posterior superior iliac spine of patients in a heparin sodium anticoagulation tube and was processed.

### HEMSC and hBMSC cultivation and identification

Single cells derived from endometrium were obtained, as previously published [14, 15]. Briefly, the endometrium was minced and suspended in PBS, containing collagenase type 3 (300 μg/ml; Worthington Biochemical Corp, Freehold, NJ) and 40 μg/ml of deoxyribonuclease type I (Worthington Biochemical Corp.). The tissue was then digested for 45 min with gentle shaking (37 °C, 110 rpm). The dissociated cell suspension was filtered through a 40 μm cell strainer, and the single-cell suspension was neutralized with DMEM/F12, containing 10% FBS in an equal volume. The undigested tissue was further digested, and cell suspension was combined and cultured, as described in previous studies [16]. Cells were expanded via passaging 3-6 times for subsequent experiments.

HEMSC and hBMSC characteristics were identified by flow cytometry. HEMSCs or hBMSCs (1 × 10^6^ cells/ml) from each sample were incubated with cell surface marker antibodies or isotype-identical IgG control (FITC Mouse Anti-Human CD90, Cat #: 51-9007657; PE Mouse Anti-Human CD44, Cat #: 51-9007656; APC Mouse Anti-Human CD73, Cat #: 51-9007649; PerCP-CyTM5.5 Mouse Anti-Human CD105, Cat #: 51-9007648; PE hMSC Negative Cocktail, Cat #: 51-9007661; all from BD Biosciences) for 30 min on ice. Cells were centrifuged and washed after each incubation period and examined using a Becton Dickinson LSRII flow cytometer.

### HEMSC proliferation, migration and differentiation

The proliferation characteristics of hEMSCs was evaluated and compared with hBMSCs using BrdU (5-Bromo-2′-deoxyuridine, Cat#: B5002, Sigma) pulse chasing and cell counting to construct the growth curve. For the BrdU assay, cells were incubated with a medium containing BrdU (10 μM) for 24 h, then washed by PBS, and fixed with 4% paraformaldehyde for 30 min at room temperature. After denaturing the DNA in 2 N HCl (10 min at 37 °C), the cell membranes were permeabilized with Triton x-100 (0.1%), and non-specific binding sites blocked with 5% bovine serum albumin. The cells were stained with a rat anti-BrdU antibody (Cat#: ab6326, 1:100, Abcam) overnight at 4 °C. After incubation with the primary antibody, the cells were washed three times with PBS and incubated with an Alexa Fluor 546 conjugated goat anti-rat antibody (Cat#: A11081, 1:50, Invitrogen) for 2 h at room temperature. The nuclei were stained with 4′, 6-diamidino-2-phenylindole (DAPI, Sigma, Cat#: D9542). BrdU-positive cells were counted by ImageJ software, and the percentage of such cells was calculated. To evaluate cell growth, hEMSCs and hBMSCs were each seeded in a 24-well plate, with 7000 cells/well. All cells were plated in triplicate for each time point. Cell numbers were counted every 2 days for up to 8 days, and the growth curve was established.

To determine the migratory potential of hEMSCs, wound healing cell migration assay was performed. HEMSCs and hBMSCs were both cultured in their respective 6-well plates. Cells were scratched with a pipette tip when confluence level approached ~ 100%, washed twice with PBS and incubated in serum-free medium for 24 h at 37 °C. Images were taken by an inverted microscope (Nikon, Japan), and the migration rate was measured and analyzed by ImageJ software.

To examine the differentiation ability of hEMSCs, cells were cultured in a specific differentiation-induction media (differentiation-induction media shown in Table S1) for 24 (adipogenic differentiation) or 12 days (osteogenic differentiation). Media were changed every 3 days, for both control and differentiation-induction media. After the time period for adipogenic or osteogenic differentiation was completed, cells were fixed with 4% paraformaldehyde, washed with PBS for 3 times, and then stained by Oil Red O for lipid droplets, as well as alizarin red for calcium crystal deposition. Pictures were taken by an inverted microscope. The percentage of differentiation rate was measured and analyzed by ImageJ software.

### Matrigel tubule assay

The pro-angiogenic potential of hEMSCs was assessed in vitro by Matrigel tubule formation, using conditioned medium and compared to the hBMSC conditioned medium. Human umbilical vein endothelial cells (HUVECs) were used in the assay to form the tubular structure. In brief, growth factor-reduced Matrigel (Becton Dickenson) was thawed and placed into 96-well (100 μl/well) culture plates at 37 °C for 30 min to allow solidification. HUVECs were seeded into coated wells at 3.5 × 10^4^ cells/well with control, hEMSC-, or hBMSC-conditioned medium. Cells were incubated for 3 h. Pictures were then taken by a Nikon microscope, and the numbers of tubes formed were counted to measure tubule-forming ability.

### In vivo rat myocardial infarction model using hEMSCs and hBMSCs

Female nude rats from Charles River Laboratories (180–250 g at 8 weeks old) were used for the procedures. All animal procedures were approved by the Animal Care Committee of the Shanxi Medical University. All experiments were carried out in accordance with the Guide for the Care and Use of Laboratory Animals (NIH, 8th Edition, 2011).

All the female nude rats were anesthetized with 2% isoflurane gas. The left anterior coronary artery was permanently ligated (LAD) by 7-0 silk suture to induce MI. One week after the first operation, rats with MI were randomized into three experimental groups: control group receiving PBS injection (PBS control), hBMSC, and hEMSC groups. Red fluorescent protein (RFP)-transduced hBMSCs or hEMSCs (3 × 10^6^/rat), both within a final volume of 100 μl, respectively, were injected into the ischemic border zone at three sites. Control rats received the same volume of PBS without cells.

### Echocardiography

The echocardiograph was measured at baseline, as well as at 0, 7, 14, 21, and 28 days after cell implantation. Left ventricular internal diameter end systole (LVIDs) and end diastole (LVIDd), as well as ejection fraction (EF %) were measured from M-mode images obtained by left ventricular short-axis view.

### ^18^F-FDG microPET/CT imaging

Images were taken at the supine position by the IRIS micro-PET/CT scanner (French, Inviscan). ^18^F-FDG was automatically synthesized using a Multi-functional Composite Module (F300Ek, Sumitomo Heavy Industries, Ltd, Japan). MicroPET imaging was performed for 20 min at baseline, as well as at 0 and 28 days after cell implantation. The nude rats were anesthetized with 2% isoflurane and injected with ^18^F-FDG (approximately 7 MBq dose) through the tail vein. Imaging acquisition was started at 40 min after ^18^F-FDG administration, and the animals were restrained in a scan bed to prevent any movement during the scanning period. CT images were first acquired, followed by 10 min static PET scanning in the heart area. All image analyses were performed by PMOD version 4.0 image analysis software (PMOD Technologies Ltd., Zurich, Switzerland). Reconstructed PET data were reoriented into 17 segments of polar map images, and each polar map was normalized to its maximum.

### Masson’s trichrome staining

After functional analyses were completed (28 days after cell implantation), the hearts were perfused with PBS and dissociated from surrounding tissue. Following fixation with 10% formalin for 48 h and dehydration with 75% ethanol, the hearts were then cut along the horizontal axis into continuous 1-mm sections and photographed for morphometry. The size of the infarcted area, defined as the ratio (percentage) of scar length to the entire left ventricular circumference, as well as the scar thickness, were measured by ImageJ software. After planimetry, the heart segments were embedded into paraffin and sectioned at 10 μm thickness. Masson's trichrome staining was performed to confirm the presence of scar tissue in the left ventricular free wall (red staining represented viable cardiac tissue, and blue represented collagen fibers in the scarred tissue), as described by the manufacturer’s specifications (Sigma).

### Immunofluorescent staining

Heart sections were immuno-labeled with antibodies against RFP, α-smooth muscle actin (α-SMA), and isolectin. The slides were incubated with primary antibodies (mouse anti-α-SMA, Sigma, Cat#: A5228 at 1:800; rabbit anti-RFP, Abcam, Cat#: ab62341 at 1:200) at 4 °C overnight. The next day, incubation with respective Alexa 488 or 546 conjugated secondary antibodies (Invitrogen) at 1:2000, or Isolectin GS-IB4 488 conjugate (Invitrogen, Cat#: I21411) at 1:50, was carried out at room temperature, with protection from light, for 1 h. Nuclei were stained with DAPI. The fluorescent-positive area in three randomly selected high-power fields per slide was measured and averaged with a Nikon fluorescence inverted microscope.

### RNA and real-time qPCR

Total RNA was isolated from cells or heart tissue (scar and border zones) using TRIzol reagent (Invitrogen), following the manufacturer’s instructions. One μg total RNA served as the template for cDNA synthesis with SuperScript TM Reverse Transcriptase IV (Invitrogen). TB Green® Premix EX Taq II (TaKaRa) was used to detect the accumulation of PCR products during cycling with the ABI Real-time PCR Step One Plus (Applied Biosystems). All reactions were carried out in triplicate. Fold differences in the expression level of each gene were calculated using CT values and normalized to the housekeeping gene GAPDH. All primers were designed using qPCR assay design software (Integrated DNA Technology), and their sequences were provided in Table S2.

### Protein extraction and Western blotting

To determine protein levels, total protein was extracted from cells or heart tissue (scar and border zones), and standard protocols were followed, as previously described [17]. As for antibody usage, β-actin (sc-47778, Santa Cruz Biotechnology, 1:2000) was used as the loading control. Involved antibodies included VEGFA (Cat#: ab1316, Abcam, 1:1000), Angiopoietin 1 (ANG1, Cat#: ab183701, Abcam, 1:10000), Angiopoietin 2 (ANG2, Cat#: ab155106, Abcam, 1:2000), Fms-like tyrosine kinase 1 (FLT-1, Cat#: 13687-1-AP, Proteintech, 1:1000), TIE2 (Cat#: 19157-1-AP, Proteintech, 1:1000), FGF9 (Cat#: ab206408, Abcam, 1:1000), and TGFβ2 (Cat#: ab113670, Abcam, 1:900)

### Statistical analysis

Data are expressed as mean ± SEM. Statistical analyses were performed using GraphPad Prism 7 and SAS 9.2 software. The Shapiro-Wilk test was used to test the normality of the data. All data exhibited a normal/Gaussian distribution. The unpaired Student’s t test (2-tailed) was performed for comparisons between two groups, while comparisons of parameters among three or more groups were analyzed using either one-way analysis of variance (ANOVA) or two-way ANOVA with repeated measures over time, followed by, respectively, Tukey’s or Bonferroni post hoc tests. Differences were considered statistically significant at *P* < 0.05.

## Results

### Isolation and characterization of hEMSCs

**Fig. 1:**
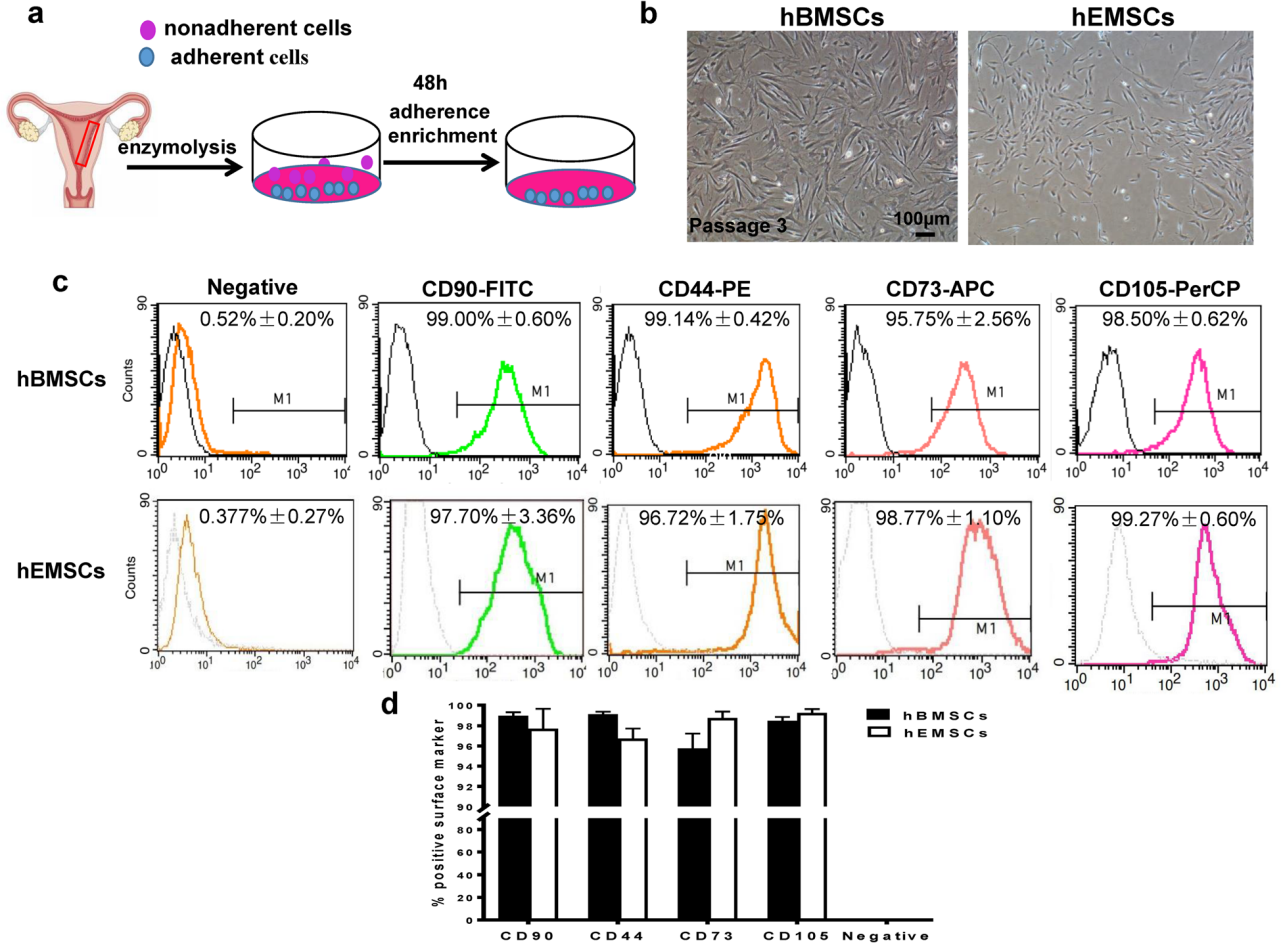
Cultivation and identification of hEMSCs. **a** Schematic illustration of isolating and culturing of human endometrium-derived stem cells (hEMSCs). **b** Representative phase-contrast morphological observation of human bone marrow mesenchymal stem cells (hBMSCs) and hEMSCs cultured up to passage 3 after isolation from premenopausal donors (30–50 years old). **c** Representative histogram plots of cell surface markers from hEMSCs and hBMSCs. **d** Comparison of % positive cells of hEMSCs and hBMSCs. Data are expressed as mean ± SEM. *n* = 3/group

HEMSCs were successfully isolated using the procedure outlined in Fig. 1a. At the third generation, the endometrium-derived cells were smaller in size than the hBMSCs (Fig. 1b). To characterize the hEMSCs, flow cytometry was performed to evaluate the cell surface markers for mesenchymal and hematopoietic stem cell lineages. The data showed that these cells were positive for mesenchymal stem cell markers CD90, CD44, CD73, and CD105, but negative for hematopoietic lineage cell markers CD45, CD11b, CD19, and HLA-DR (Fig. 1c). Quantification of the percentage of positive cells for these surface markers showed both hEMSCs and hBMSCs being > 90% positive for CD90, CD44, CD73, and CD105, and no significant difference between those cell types for these markers existed (Fig. 1d). These results suggested that hEMSCs had a similar phenotype as hBMSCs, thereby meeting the criteria identifying them as mesenchymal stem cells.

### Proliferative, migratory, and differential potentials for hEMSCs

**Fig. 2:**
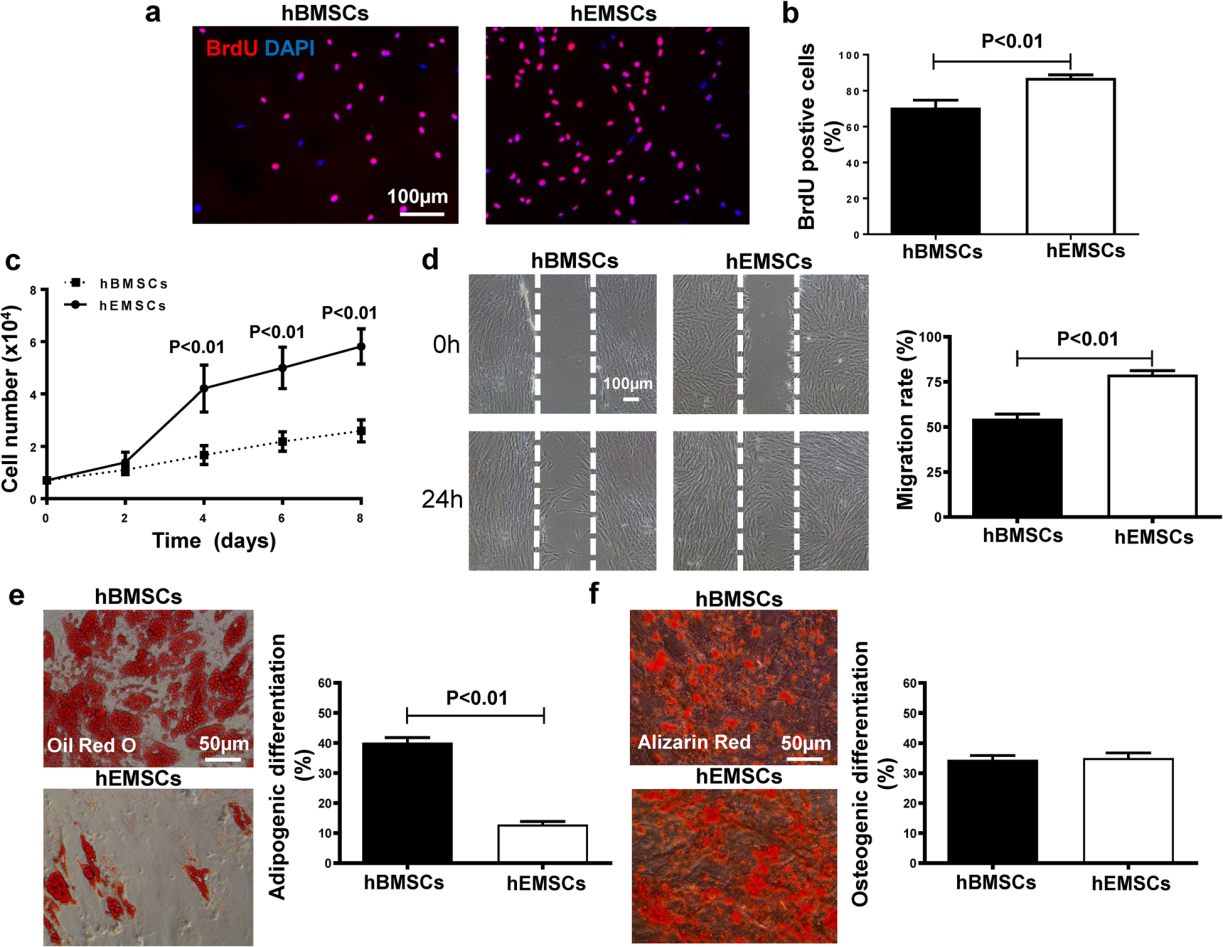
Functional analyses of hEMSCs. **a**, **b** Cell proliferation was assessed by 5-Bromo-2′-deoxyuridine (BrdU) pulse chasing. Representative micrographs of immunofluorescent staining of BrdU (red), nuclei stained with 4′, 6-diamidino-2-phenylindole (DAPI, blue), and quantification of the percentage of BrdU^+^ cells in hEMSCs vs hBMSCs. **c** The rate of cell growth was significantly higher in hEMSCs vs hBMSCs. **d** Cell migration was assessed by wound-scratch assay. Representative images showed the cell migration of hEMSCs and hBMSCs, and migration rate was significantly higher in hEMSCs. **e** Representative micrographs of adipogenic differentiation by Oil Red O staining of lipid droplets. The adipogenic differentiation rate was significantly lower in hEMSCs vs hBMSCs. **f** Representative micrographs of osteogenic differentiation by alizarin red staining of osteocytes. The osteogenic differentiation rate was similar between hEMSCs and hBMSCs. Data are expressed as mean ± SEM. *N* = 6/group for all experiments

To further characterize hEMSCs, BrdU pulse chasing and constructing the cell growth curve were performed. Compared to hBMSCs, the percentage of BrdU^+^ cells was significantly higher in hEMSCs at 24 h after BrdU labeling (Fig. 2a, b). Consistent with the observation from BrdU labeling, the cell growth curve showed that the cell number from the hEMSC group was significantly higher than for the hBMSC group, starting from day 4 up to day 8 of the culturing period (Fig. 2c). To determine the migration ability of hEMSCs, the wound healing cell migration assay was performed, where the migration rate of hEMSCs was found to be significantly higher than for hBMSCs (Fig. 2d).

Adipogenic and osteogenic differentiation are able to be induced in hEMSCs, and a large number of cells were stained by Oil Red O (Fig. 2e) and alizarin red (Fig. 2f), confirming that these cells had adipogenic and osteogenic differentiation abilities. However, compared to hBMSCs, the adipogenic differentiation ability was significantly lower in hEMSCs, while similar osteogenic differentiation ability was present between hEMSCs and hBMSCs. These data suggested that hEMSCs had greater proliferative and migratory capacities, whereas hBMSCs had greater adipogenic differentiation capabilities.

### In vitro pro-angiogenic potentials for hEMSCs

**Fig. 3:**
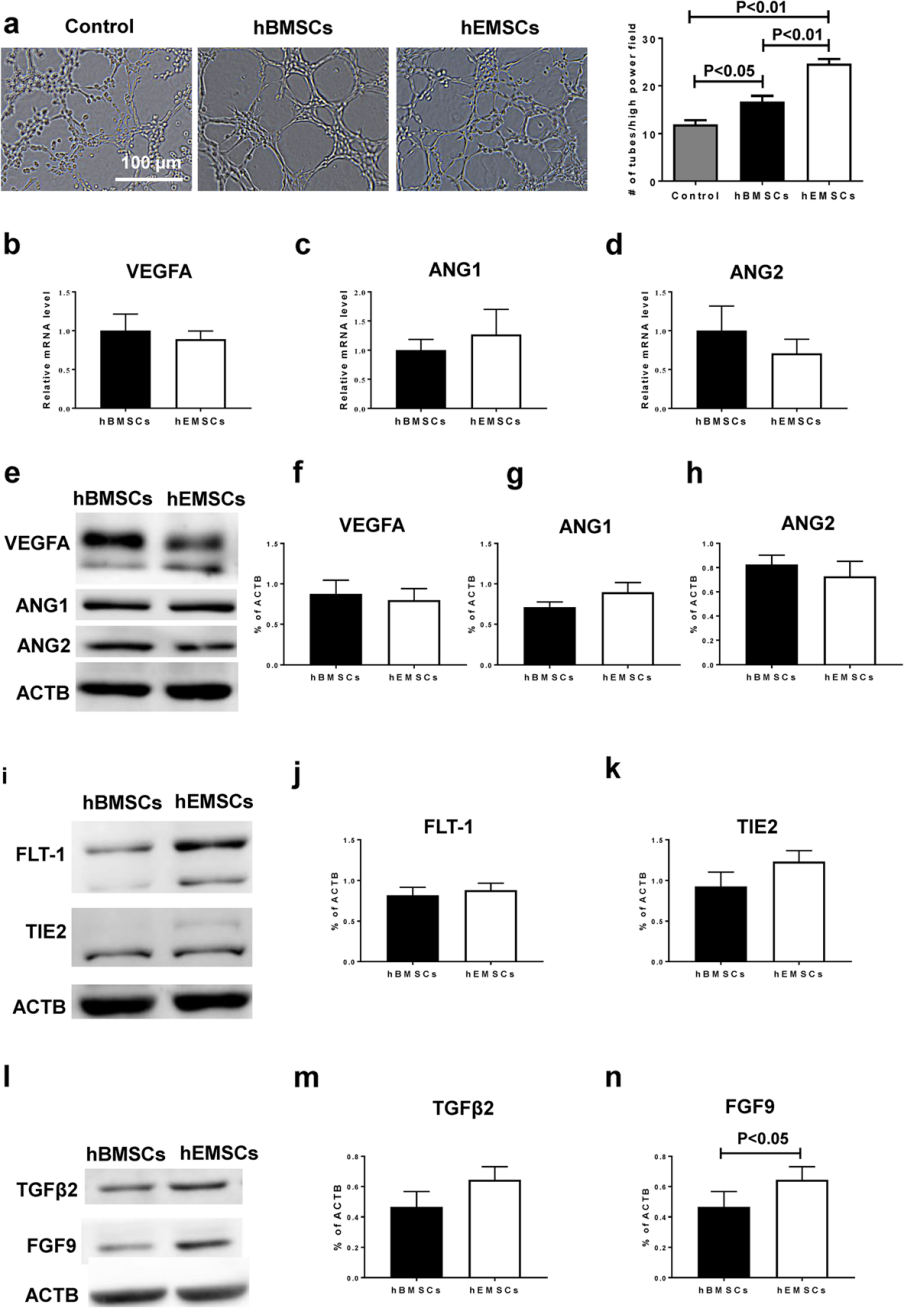
Pro-angiogenic effects of hEMSCs in vitro. **a** Representative micrographs from the Matrigel tube formation assay. The number of tubes formed was significantly higher in hEMSCs than hBMSCs, *n* = 6/group. Real-time qPCR and Western blotting were performed to measure the level of mRNA and protein expression of VEGFA (**b**, **e**, **f**), angiopoietin 1 (ANG1, **c**, **e**, **g**), and angiopoietin 2 (ANG2, **d**, **e**, **h**). Representative Western blot images (**e, i, l**) and quantification of VEGFA (**f**), ANG1 (**g**), ANG2 (**h**), Fms-like tyrosine kinase 1 (FLT-1, **j**), TIE2 (**k**), transforming growth factor β2 (**m**), and FGF9 (**n**) protein levels in hEMSCs or hBMSCs. Data are expressed as mean ± SEM. *n* = 5 or 6/group for all the real-time qPCR and Western blotting quantifications

To investigate the pro-angiogenic potentials of hEMSCs, we performed the Matrigel tubular formation assay. HUVECs were treated with either hEMSC or hBMSC-conditioned media, and the number of tubes formed was assessed. The result showed that HUVECs treated with the conditioned medium from hEMSCs had significantly higher number of tubes formed than that from hBMSCs or the control medium (Fig. 3a).

To investigate the pro-angiogenic effects of hEMSCs at the molecular level, RNA-seq analyses and protein arrays were performed for both hEMSCs and hBMSCs (Fig. S1). The number of differentially expressed genes (DEGs) between hBMSCs and hEMSC was 4576 in total, among which the number of upregulated genes and down-regulated genes in hEMSCs, compared to hBMSCs, were 2382 and 2194, respectively (Fig. S1a). After DEG analysis, 20 categories with significant enrichment were screened for functional enrichment analysis of gene ontology (GO), among which 113 genes were involved in angiogenesis (Fig. S1b). In the clustering heat map, there were significant differences in the levels of angiogenesis-related gene expression between the two cell groups (Fig. S1c). Additionally, the results of protein arrays for cell culture medium, from hEMSCs and hBMSCs, showed a total of 38 differentially expressed proteins between the supernatants obtained from hBMSCs and hEMSCs. Among the 38 differentially expressed proteins, there were 5 angiogenesis-related factors, whose data overlapped with the RNA-seq data. Of those 5 factors, PGF and ANG4 were up-regulated in hBMSCs, while FLT-1, TEK (also known as TIE2), and FGF9 were upregulated in hEMSCs. (Fig. S1d). To further validate the results from RNA-seq and protein arrays, real-time qPCR and Western Blotting analyses in both hEMSCs and hBMSCs were carried out to measure mRNA and protein expression levels for the pro-angiogenic factors: VEGFA (Fig. 3b, e, f), ANG1 (Fig. 3c, e, g), ANG2 (Fig. 3d, e, h), FLT-1 (Fig. 3i, j), TIE2 (Fig. 3i, k), transforming growth factor β2 (TGFβ2, Fig. 3l, m), and fibroblast growth factor 9 (FGF9, Fig. 3l, n). The data showed that mRNA expression levels of VEGFA, ANG1, and ANG2 in hEMSCs were comparable to hBMSCs, while FGF9 protein level was significantly higher in hEMSCs than in hBMSCs. This data indicated that the angiogenic potentials for hEMSCs were as potent as that associated with hBMSCs.

### HEMSC transplantation improved cardiac function and attenuated adverse remodeling after MI

**Fig. 4:**
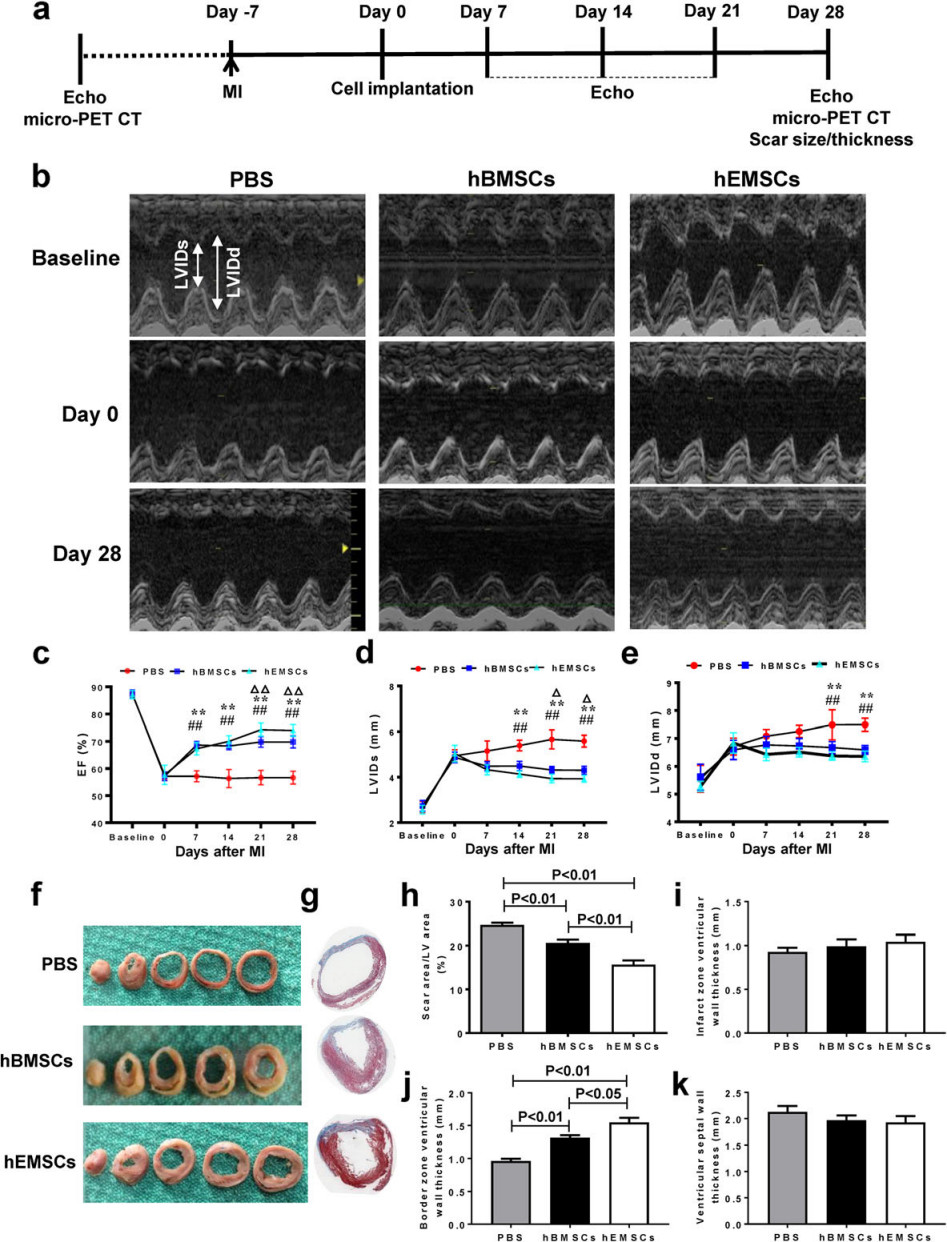
Changes in cardiac function after MI and hEMSC implantation. **a** Schematic illustration of in vivo experimental procedures. **b** Representative M-mode echocardiographic images taken before (baseline), 0 and 28 days after cell transplantation in nude rats that received injection of PBS (PBS control), human bone marrow mesenchymal stem cells (hBMSCs), or human endometrium-derived stem cells (hEMSCs). Ejection fraction (**c**), Left ventricular internal diameter end systole (LVIDs, **d**), and end diastole (LVIDd, **e**) evaluated by echocardiograph. *n* = 6/group. Representative images of heart sections (**f**) and Masson’s trichrome staining (**g**) taken at 28 days after cell transplantation. **h** Planimetry-based quantification revealed that the scar area was significantly smaller in the hEMSC group, compared to the other 2 groups at 28 days after cell transplantation. Ventricular wall thicknesses for infarct (**i**), border (**j**), and septum (**k**). Significant differences were only found for the border zone, where thicknesses were significantly higher among hEMSC compared to hBMSC groups, and both also significantly higher than the PBS control group. Data are expressed as mean ± SEM. *n* = 5/group. **p < 0.01 PBS vs hEMSCs, ##*p* < 0.01 PBS vs hBMSCs, ^Δ^*p* < 0.05 hBMSCs vs hEMSCs, ^ΔΔ^*p* < 0.01 hBMSCs vs hEMSCs

To examine the effects of implanted hEMSCs on cardiac repair, nude rats with coronary artery ligation were used. Cardiac function was determined by echocardiography pre- and post-injection of PBS control, hBMSCs, or hEMSCs (Fig. 4a). Representative M-mode echocardiographic images were obtained before (baseline), as well as at 0 and 28 days after cell transplantation (Fig. 4b). No differences between parameters among the three experimental groups at baseline were present, as determined by echocardiography (Fig. 4c, d, e). At 7 days after cell implantation, EF (Fig. 4c) was significantly higher in hEMSCs and hBMSCs groups than the PBS control group. On the other hand, LVIDs and LVIDd were lower in hEMSC and hBMSC groups, compared to the PBS control group. The same trend was sustained for up to 4 weeks after cell implantation for all 3 groups. Comparing the hBMSC and the hEMSC groups, EF was higher, whereas LVIDs was lower, in hEMSCs, compared to hBMSCs, at day 21 and 28-post cell injection, respectively (Fig. 4c, d, e). These results indicated that compared to hBMSCs, hEMSCs had better capacity with respect to restoration of cardiac function in infarcted nude rat hearts. Morphological analysis and Masson’s trichrome staining of hearts indicated that scar size (Fig. 4f, g, h) was significantly lower in the hEMSC group, compared to the other 2 groups. With respect to ventricular wall thicknesses, significant increases were only found for the border zone (infarction penumbra), where it was significantly higher among hEMSC compared to hBMSC groups, and both were also significantly higher than the PBS control group. However, no such differences were found for infarct and septal wall (remote field) thicknesses for any of the 3 groups (Fig. 4i, j, k). These data suggested that hEMSCs had a beneficial effect on adverse cardiac remodeling after MI.

### HEMSCs improved cardiac metabolism at the injured myocardium

**Fig. 5:**
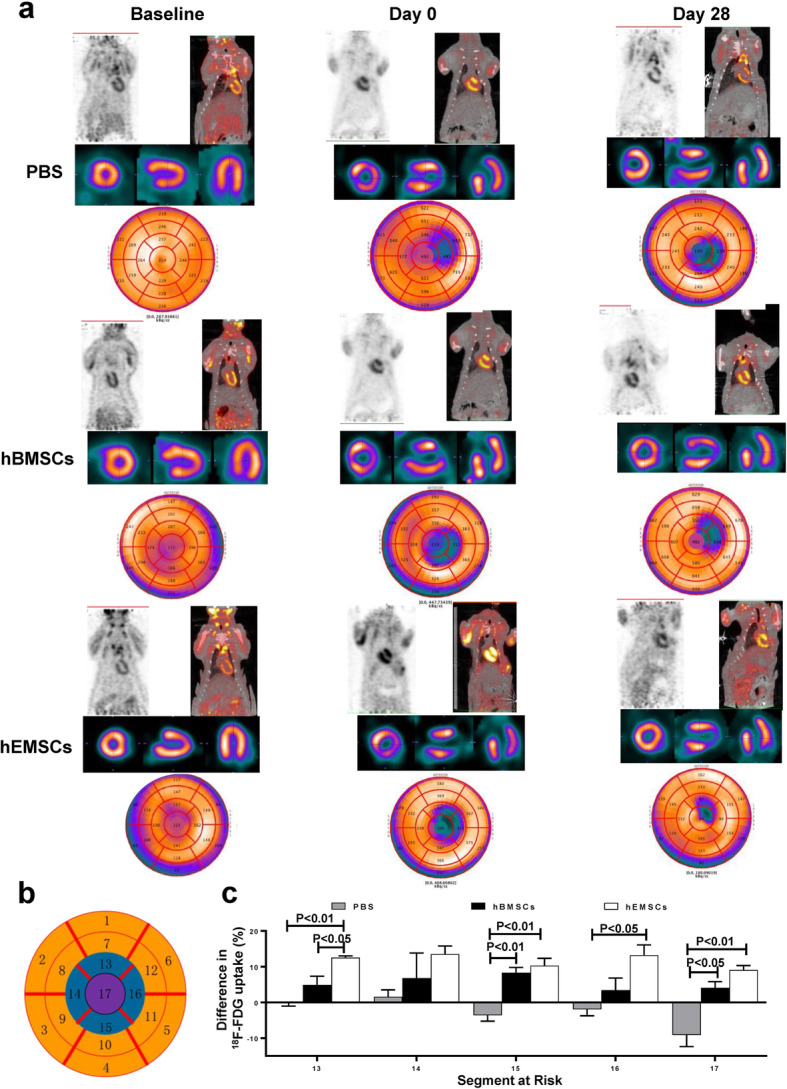
Changes in cardiomyocyte glucose metabolism after MI and hEMSC implantation. Cardiomyocyte glucose metabolism was assessed by ^18^F-FDG uptake using microPET at baseline, and at 0 and 28 days after cell transplantation. **a** Representative transverse, coronal and sagittal ^18^F-FDG uptake images in nude rats that received injection of PBS (PBS control), human bone marrow mesenchymal stem cells (hBMSCs), or human endometrium-derived stem cells (hEMSCs). **b** A pie-shape heart map was used for analysis of cellular metabolism. **c** Quantification of ^18^F-FDG uptake in the apical regions among the three groups. The increase in the uptake of ^18^F-FDG in the apical regions was highest in the hEMSC group. Data are expressed as mean ± SEM. *n* = 3/group

To examine the effects of implanted hEMSCs on myocardial metabolism after MI, we used ^18^F-FDG microPET to assess cardiomyocyte activity at baseline (-7 days), as well as at 0 and 28 days after cell implantation. Figure 5a showed representative transverse, coronal, and sagittal ^18^F-FDG micro-PET images taken before (baseline), 0, and 28 days after cell transplantation among the three groups. To evaluate regional cellular metabolism, a pie-shape heart map was used for analysis, and the heart was divided into 17 segments (Fig. 5b). Since segments 1–12 were found to not be at risk, only segments 13–17 were used for analyses and comparisons. At 7 days after MI (day 0), ^18^F-FDG uptake decreased in the apical infarction area among all three groups (Fig. 5a). However, at 28 days after cell transplantation, ^18^F-FDG uptake at the apical infarction area was significantly higher in both hBMSC and hEMSC groups, compared to the PBS control group, with the highest uptake being present in the hEMSC group (Fig. 5c). HEMSC implantation improved regional cardiac metabolism ~ 3–15 times compared to the PBS control group, and 2–3 times compared to hBMSCs, at the defective segments. These data suggested that hEMSC treatment improved cardiomyocyte metabolism, which may have resulted from more surviving cardiomyocytes being present after MI.

### HEMSC transplantation increased angiogenesis after MI

**Fig. 6:**
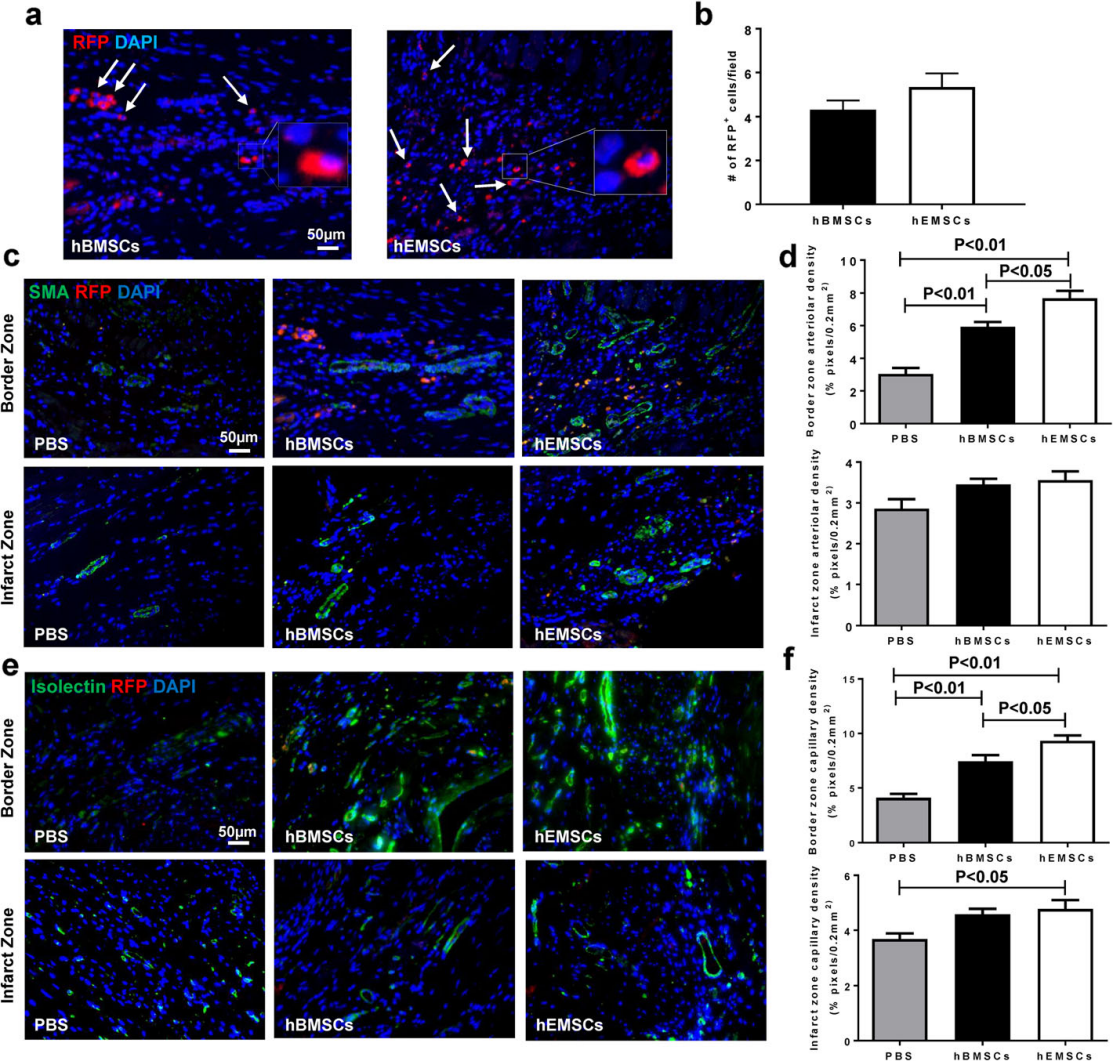
In vivo evaluation of survival and angiogenesis of transplanted hEMSCs after MI. **a** Representative micrographs of heart sections showing surviving transplanted cells in hBMSC and hEMSC groups at 28 days after cell injection. Transplanted cells stained with red fluorescent protein (RFP), nuclei stained with 4′, 6-diamidino-2-phenylindole (DAPI). **b** Quantification of the number of the survived transplanted cells in the infarcted region. **c**, **d** Representative micrographs of heart sections, from border and infarct zones, showing arteriole densities (stained with α-smooth muscle actin, green) being significantly higher in hEMSCs, compared to hBMSC and PBS control, in the border zone. **e**, **f** Representative micrographs of heart sections, from border and infarct zones, showing capillary densities (stained with isolectin, green) being significantly higher in hEMSCs, compared to hBMSCs and PBS control, in the border zone. Data are expressed as mean ± SEM. *n* = 5 or 6/group for all the experimental groups

To assess implanted cell survival, RFP^+^ cells were quantified at the infarcted area at 28 days post-cell transplantation. The number of RFP^+^ cells at the implanted area was comparable between hEMSC and hBMSC groups, indicating the cell survival rate being similar between the two groups (Fig. 6a, b). To assess blood vessel formation, arteriole and capillary densities were quantified by α-SMA and isolectin staining, respectively [18]. Arteriolar density was significantly higher among the hEMSC group compared to hBMSC in the border zone of the infarct, and both were also significantly higher than the PBS control group. However, no significant differences were present among the three groups with respect to the infarct zone (Fig. 6c, d). The same trend was observed in the border zone for the capillary densities, while in the infarct zone, capillary density was significantly higher for only the hEMSC group versus PBS control (Fig. 6e, f). Overall, compared to the hBMSC group, capillary and arteriolar densities were significantly increased in the hEMSC group, indicating stronger angiogenic capacity in the hEMSC group. To investigate whether hEMSCs had the potential to differentiate into endothelial or smooth muscle cells directly involved in forming vascular structures, dual immunofluorescent staining with RFP and isolectin, or α-SMA, was performed to localize RFP/isolectin or RFP/α-SMA double positive cells (Fig. 6c, e). No a-SMA^+^ hEMSCs were observed at 28 days after cell transplantation (Fig. 6c). However, low numbers of RFP^+^/isolectin^+^ hEMSCs were detected, suggesting the possibility of low-level endothelial-like differentiation. However, the RFP^+^/isolectin^+^ cells were not integrated into the vasculature (Fig. 6e). These data implied that hEMSCs probably promoted angiogenesis more through paracrine action than direct cell differentiation.

### Angiogenic molecules were involved in modulating improvement in heart function after hEMSC transplantation

**Fig. 7:**
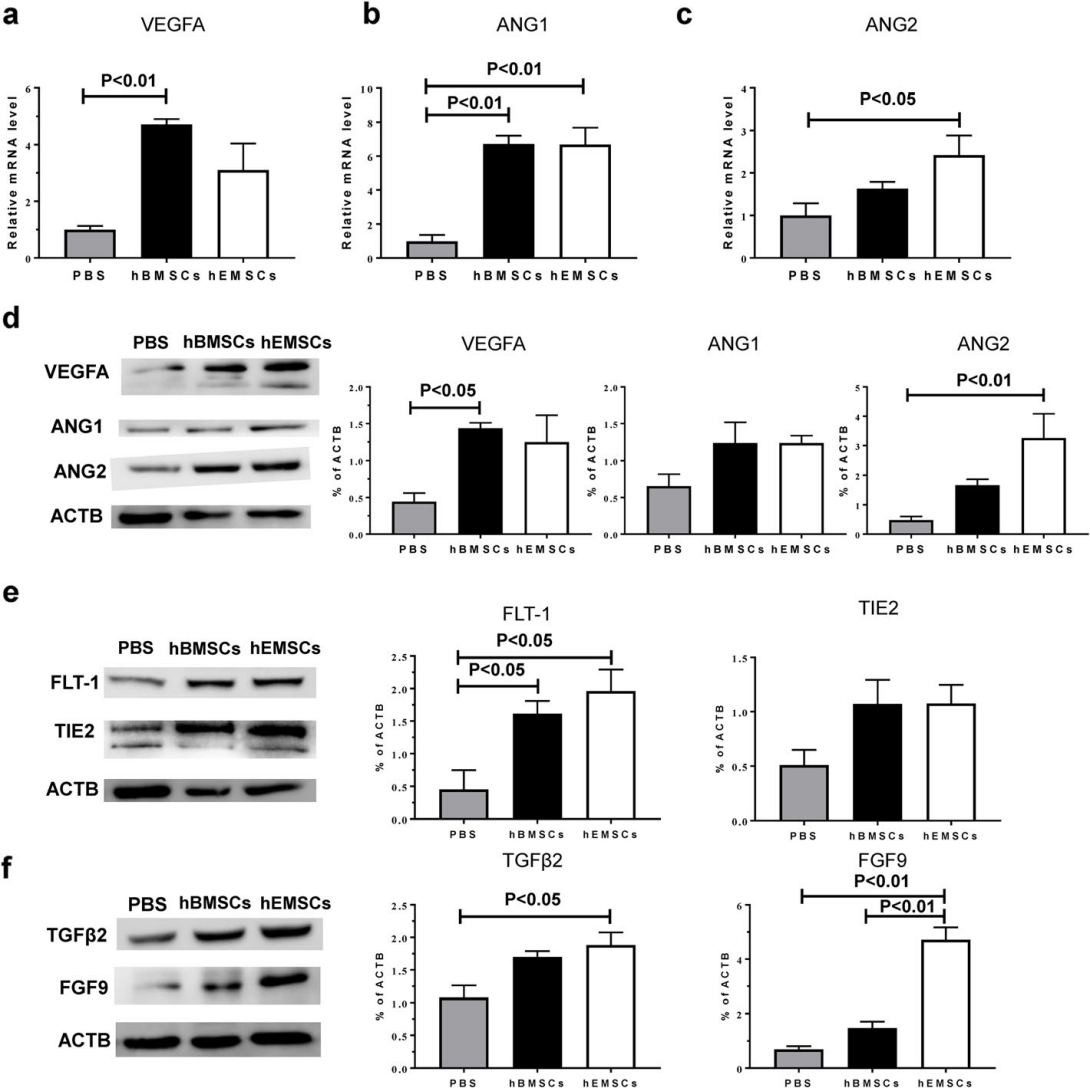
In vivo implantation of hEMSCs enhanced the expression of pro-angiogenic cytokines after MI. The level of mRNA expression for VEGF (**a**), angiopoietin 1 (ANG1, **b**), and angiopoietin 2 (ANG2, **c**) was measured in the infarcted heart tissue of nude rats at 3 days after cell transplantation by real-time qPCR. Individual values were normalized to glyceraldehyde 3-phosphate dehydrogenase (GAPDH). Representative Western blot images and quantification of VEGFA, ANG1 and ANG2 (**d**), Fms-like tyrosine kinase 1 (FLT-1) and TIE2 (**e**), transforming growth factor β2 and FGF9 (**f**) protein levels in the infarcted heart tissue of nude rats at 3 days after cell transplantation. Data are expressed as mean ± SEM. *n* = 3 or 4/group for all assays

To elucidate the possible mechanisms of hEMSC therapy on modulating heart function improvements, mRNA expression of cytokines known to promote angiogenesis, including VEGFA (Fig. 7a), ANG1 (Fig. 7b), and ANG2 (Fig. 7c), were measured in the infarct zone at 3 days after cell transplantation. Real-time qPCR results showed an increasing trend in both hBMSC and hEMSC groups, compared to the PBS control group (Fig. 7a, b, c). In addition, protein expression of cytokines known to promote angiogenesis, including VEGFA, ANG1, ANG2 (Fig. 7d), FLT-1 and TIE2 (Fig. 7e), as well as TGFβ2 and FGF9 (Fig. 7f), were measured in the infarct zone at 3 days after cell transplantation. Western blotting results showed that hEMSCs significantly upregulated the expression of three angiogenesis-related factors, ANG2, FLT-1, and FGF9, in comparison with the PBS control group. Although the other angiogenesis-related factors showed no statistical significance, there was an increasing trend in both hBMSC and hEMSC groups, compared to the PBS control group. These data suggested that hEMSCs promoted cardiac repair, probably through augmenting the expression of multiple biological factors associated with enhancing angiogenesis.

## Discussion

Despite multiple cellular types demonstrating great potency to improve cardiac function, the application of cell therapy faces many obstacles, owing to their rarity in tissues necessitating for in vitro expansion, as well as donor age affecting their quality and capacity for tissue repair [19, 20]. In this study, we successfully isolated a population of highly pro-angiogenic cells derived from human endometrium and evaluated their capabilities as a cell source for cardiac repair after MI. In vitro, we carefully characterized these cells and found they were positive for mesenchymal stem cell markers of CD90, CD44, CD73, and CD105, but negative for hematopoietic lineage cell markers of CD45, CD11b, CD19, and HLA-DR, meeting the criteria for being mesenchymal stem cells. We compared the proliferative, migratory, differentiation, and angiogenic capacities of age-matched hEMSCs with hBMSCs and found that hEMSCs had greater proliferative, migratory and angiogenic capacities, with higher expression levels of some angiogenic factors, such as FGF9. In vivo hEMSC transplantation preserved cardiac function, decreased infarct size, and improved repair post-MI. Mechanistically, angiogenesis is mainly responsible for improvements in cardiac function. Indeed, we showed that these highly angiogenic cells were associated with higher expression of factors involved in promoting and sustaining neovascularization, as well as the preservation of viable myocytes to support tissue repair in vivo*.*

The human endometrium of premenopausal women undergoes repeated cycles of regeneration, differentiation and shedding. This remarkable self-renewal capacity thus suggests that the endometrium contains adult stem/progenitor cells [21]. These cells have been successfully isolated and identified from human and mouse endometria [14, 15, 22]. Our group also demonstrated the presence of stem/progenitor cells in the mouse endometrium, which can differentiate into blood and/or endothelial progenitor cells [23]. In this study, we isolated stem/progenitor cells derived from human endometrium by mechanical and enzymatic approaches. The hEMSCs derived from premenopausal endometrium share the surface marker phenotypes with hBMSCs and fulfill the criteria defining MSCs, such as self-renewal and multipotency. These results suggested a successful isolation of a population of stem cells closer to mesenchymal lineages. In our study, the heterogeneity of the cell fraction may have contributed to multiple mechanisms of improvement in cardiac function: higher angiogenesis counteracting ischemic injury, along with better myocyte survival resulting in smaller scar size and modulation of ventricular remodeling [24, 25].

One of the required characteristics for an ideal cell source for clinical treatment is having strong proliferation abilities. In this study, we compared the proliferation capacity of age-matched hEMSCs and hBMSCs. The result revealed that hEMSCs have a stronger proliferative capacity, consistent with previously reported results [26–28] from studies on mesenchymal stem cells derived from menstrual blood. Stronger proliferative capacities for stem or progenitor cells are associated with better repair functions. In addition, tissue repair also involves migration properties. The wound healing cell migration assay demonstrated that the migration ability of hEMSCs was higher than hBMSCs, further supporting the superior repair ability of hEMSCs. On the other hand, we have observed some variation in the levels of differentiation abilities of hEMSCs compared to hBMSCs, where both cell types had similar osteogenic differentiation abilities, but hEMSCs exhibited a lower level of adipogenic potential. This variation in differentiation could be owed to differences in stem cell niches where hEMSCs are found, compared to hBMSCs, as the uterine endometrium requires greater angiogenic capabilities to support the cyclic proliferation and shedding of the endometrial layer during the menstrual cycle. One possible difference between those niches could be the presence of PPAR-γ ligands within the hBMSC niche, as the activation of their corresponding PPAR-γ receptors is considered to be a key factor for adipocyte differentiation [29]. By contrast, those ligands may be less present in the hEMSC niche, as excess PPAR-γ activation, through its subsequent stimulation of IL-6, can contribute to the onset of endometriosis in the female reproductive system [30]. Therefore, this result implies that hEMSCs may possess the ability to differentiate into some cellular types other than those that can be differentiated from hBMSCS. Indeed, some studies have shown that mesenchymal stem cells derived from menstrual blood or endometrium are more capable of differentiating into cardiomyocytes, endothelial cells, and neurons [26, 31, 32], indicating a possibly superior spectrum of hEMSC differentiation potentials. In particular, one study showed that CD105^+^ porcine EMSCs were able to differentiate toward cardiomyocyte-like cells, which was confirmed through the expression of cardiomyocyte lineage-specific marker genes, such as DES, ACTA2, cTnT, and ACTC1 [33]. Along these lines, Hida et al. successfully differentiated human menstrual blood-derived MSCs, the freely-shed EMSCs, into spontaneously beating cardiomyocyte-like cells through co-culture with murine cardiac cells, where those MSCs expressed troponin I and connexin 43, as well as characteristic contractile-associated striations of intermediate filament α-actinin [34]. These differentiated MSCs also showed pacemaker and action potentials potential characteristic of cardiomyocytes under electrophysiological studies. Implantation of EGFP-tagged human menstrual blood-derived MSCs into infarcted mouse hearts improved left ventricular systolic functions, as well as showing in vivo spontaneous cardiomyocyte-like differentiation. These menstrual blood MSC findings were also supported by Rahimi et al., who showed that those cells, either when treated with 5-aza-2′-deoxycytidine and bFGF, or co-cultured with native cardiomyocytes, are able to differentiate into cardiomyocyte-like cells, as judged by the expression of connexin-43, and cardiac troponin T [35, 36]. It is worth noting that these studies only demonstrated the tendency of those EMSCs to differentiate into cardiomyocyte-like cells, which are still far from being mature cardiomyocytes with fully functional electrophysiological and contractile characteristics.

However, in our study, although hEMSCs showed some in vitro osteogenic and adipogenic differentiation ability, we failed to find any in vivo hEMSC differentiation into endothelial and smooth muscle cell lineages, at least during the 28-day study period. Based on the in vivo findings, we postulated this is the same case for cardiomyocyte differentiation. We believe that in our case hEMSCs participate to the cardiac repair process more through angiogenic effects promoting the survival of the remaining cardiomyocytes within the recipient, rather than them directly differentiating into cardiomyocytes.

Growing evidence suggests that paracrine effects play key roles in stem cell therapy [37]. To unravel the molecular basis responsible for the beneficial effects of hEMSCs, we evaluated the expression levels of factors known to enhance angiogenesis both in vitro and in vivo. In vitro, compared to hBMSCs, the FGF9 protein level was significantly higher in hEMSCs. FGF9, similar to other family members such as FGF2, has been shown to play a role in coronary neovasculogenesis and enhancing micro-vessel density in the post-MI heart [38, 39]. When comparing hEMSCs to hBMSCs in Matrigel assays, the data showed the angiogenic function of hEMSCs was superior to that of hBMSCs. These findings were consistent with the in vivo results showing that compared to the hBMSC group, arteriolar and capillary densities were significantly increased in the hEMSC group, indicating stronger angiogenic abilities. Multiple angiogenic factors were also examined in the infarcted hearts at 3 days after cell transplantation. Most of the factors activated in vitro in hEMSCs were also upregulated to levels comparable to hBMSCs in vivo. All these findings supported our hypothesis that hEMSCs are a highly angiogenic cell source, with great potential in the treatment of ischemic diseases.

Cardiomyocyte apoptosis after MI is the main cause associated with the deterioration of cardiac function. As a result, improving cardiomyocyte activity and survival is an important aspect of therapy to restore cardiac function. Cardiomyocyte glucose metabolism imaging is an efficient, noninvasive, and rapid assessment of cardiomyocyte activity. ^18^F-FDG is a glucose analog that can be transported into cells by their glucose transporters, where it is phosphorylated to ^18^F-FDG-6-phosphate, which cannot undergo further metabolism, thereby remaining trapped within the cells. Increased glycolysis or energy metabolism in cardiomyocytes may result in enhanced ^18^F-FDG uptake and accumulation, serving as an index of better cardiomyocyte activity or survival. In this study, we assessed myocyte glucose metabolism, using ^18^F-FDG microPET after MI and cell transplantation. The result showed that a significant increase in glucose metabolism at the apical infarction area was detected in the hEMSC group at 28 days after cell transplantation, which may have resulted from better survival of cardiomyocytes after MI. This finding is consistent with a previously reported result showing that mesenchymal stem cells, derived from menstrual blood, promoted cardiomyocyte activity [40]. Taken together, all evidence supported the notion that these highly pro-angiogenic cells, derived from human endometrium, preserved myocyte metabolism and survival probably through the restoration of neovascularization.

Our study, however, has several limitations. In light of possible future clinical applications, hEMSCs are most likely used in an allogenic setting for both female and male patients. Therefore, assessing their immunomodulatory properties and efficacy in male subjects are warranted. It is worth noting, though, that in our previous study for a mouse model, we found that > 20% of mouse uterus cells did not have any detectible MHC I expression [12]. Those MHC I-negative cells, when co-cultured with mixed leukocytes, had reduced cell death and leukocyte proliferation compared to MHC I-positive cells. Furthermore, under post-MI intra-myocardial injection, those cells demonstrated properties comparable to those in syngeneic bone marrow cell transplantation, with engraftment in cardiac tissue along with limited CD4 and CD8 cell recruitment. Based on those findings, we postulate that hEMSCs would share the same immuno-privilege and immunomodulatory properties as those MHC I-negative cells. Another limitation relates to the potential of tumorigenesis from hEMSCs, considering their derivation from hysterectomy patients with fibrosis or adenomyosis. However, in our study, we assessed cardiomyocyte metabolism with ^18^F-FDG microPET-CT, which could also detect the presence of tumors, and none were observed for up to 28 days after cell implantation. Nevertheless, future long-term safety studies are necessary for screening out any potential tumorigenic cells from endometrial samples before therapeutic use. Furthermore, there are some technical shortcomings in this study, such as hEMSCs being quantified by RFP labeling, rather than a more sensitive in vivo bioluminescent imaging technique better-equipped to track cell retention in live animals, as well as measuring cardiac function based on the short-axis, which is more prone to human error than the LV long-axis method [18]. Lastly, future studies with larger sample sizes should be performed to confirm the presence of key differences in protein/mRNA expression levels behind the cardio-protective effects of hEMSCs.

## Conclusions

We successfully isolated a population of highly pro-angiogenic cells derived from human endometrium and evaluated their capabilities as a cell source for cardiac repair after MI. In vitro, we carefully characterized these cells and found they were positive for mesenchymal stem cell markers, meeting the criteria of being mesenchymal stem cells. Compared to age-matched hBMSCs, hEMSCs had greater proliferative, migratory, and angiogenic capacities, with higher expression levels of FGF9. In vivo hEMSC transplantation preserved cardiac function, decreased infarct size, and improved repair post-MI. Angiogenesis was identified as the major contributor for the improvement in cardiac function, which was associated with higher expression of factors to promote and sustain neovascularization, as well as the preservation of viable myocytes. The potent angiogenic properties of hEMSCs suggest that they should be further explored as a cell therapy approach to mitigate adverse cardiovascular outcomes.

## Supplementary Information

**Additional file 1: Fig. S1.** Results from RNA-seq and Protein array. a Heatmap from RNA-seq representing expression levels for differentially expressed genes (DEGs) between hBMSCs and hEMSCs. Red: upregulation; Blue: downregulation. Expression intensity was based on R software analysis of gene expression levels. b Twenty categories with significant enrichment were screened for functional enrichment analysis of gene ontology (GO). Yellow line: Number of genes. Bars: Significance level. c Heatmap showing up- and down-regulation for DEGs in hBMSCs and hEMSCs related to angiogenesis. d Venn diagram and protein array analysis between hBMSCs and hEMSCs for DEGs. Black circle in Venn diagram represents DEGs related to angiogenesis between hEMSCs and hBMSCs by RNA-seq, while gray circle represents DEGs identified through protein arrays. The intersection of the two circles represents overlapping DEGs between RNA-seq and protein array for angiogenesis, where five angiogenesis-related factors were found, of which PGF and ANG4 were up-regulated in hBMSCs, while FLT-1, TEK (also known as TIE2), and FGF9 were upregulated in hEMSCs. FGF9: Fibroblast growth factor 9; FLT-1: Fms-like tyrosine kinase 1; PGF: Placental growth factor; ANG4: Angiopoietin 4. **Table S1.** Formulation of Differentiation Media. **Table S2.** Real-time qPCR primer sets.

## Data Availability

The datasets used and/or analyzed during the current study are available from the corresponding author on reasonable request.

## Abbreviations

^18^F-FDG: ^18^F-fluorodeoxyglucose
ANG1: Angiopoietin 1
ANG2: Angiopoietin 2
BrdU: 5-Bromo-2′-deoxyuridine
EF: Ejection fraction
FGF9: Fibroblast growth factor 9
FLT-1: Fms-like tyrosine kinase 1
hBMSCs: Human bone marrow mesenchymal stem cells
hEMSCs: Human endometrium-derived stem cells
HLA-DR: Human Leukocyte Antigen – DR
HUVECs: Human umbilical vein endothelial cells
LVIDd: Left ventricular internal diameter end diastole
LVIDs: Left ventricular internal diameter end systole
MI: Myocardial infarction
RFP: Red fluorescent protein
TGFβ2: Transforming growth factor β2
α-SMA: α-Smooth muscle actin
