# Correction: Human endometrium-derived stem cell improves cardiac function after myocardial ischemic injury by enhancing angiogenesis and myocardial metabolism

**DOI:** 10.1186/s13287-023-03255-1

**Published:** 2023-02-14

**Authors:** Xuemei Fan, Sheng He, Huifang Song, Wenjuan Yin, Jie Zhang, Zexu Peng, Kun Yang, Xiaoyan Zhai, Lingxia Zhao, Hui Gong, Yi Ping, Xiangying Jiao, Sanyuan Zhang, Changping Yan, Hongliang Wang, Ren-Ke Li, Jun Xie

**Affiliations:** 1 The Laboratory of Stem Cell Regenerative Medicine Research, Shanxi Key Laboratory of Birth Defect and Cell Regeneration, Key Laboratory of Cell Physiology of Ministry of Education, Shanxi Medical University, Taiyuan, China; 2 Shanxi Bethune Hospital, Shanxi Academy of Medical Sciences, The Third Hospital of Shanxi Medical University, Taiyuan, China; 3 The First Hospital of Shanxi Medical University, Taiyuan, China; 4 The Second Hospital of Shanxi Medical University, Taiyuan, China; 5 Key Laboratory of Molecular Imaging, Molecular Imaging Precision Medicine Collaborative Innovation Center, Shanxi Medical University, Taiyuan, China; 6 Toronto General Hospital Research Institute, University Health Network, Toronto, Canada

**Correction : Stem Cell Research & Therapy (2022) 12:344** 10.1186/s13287-021-02423-5

Following the publication of this article, the authors regretfully found two errors in the article and would like to make corrections:

In Figure 1b, the 1st image of hBMSCs was chosen incorrectly. In Figure 5a, the 2nd image in the first row (Day 0, PBS) was chosen incorrectly. This mistake occurred due to the carelessness in picking the representative images when the authors tried to compare the morphology for hBMSCs and hEMSCs (Fig. 1b), as well as ^18^F-FDG uptake between different treatments for Fig. 5a during the figure compilation process.

**Fig. 1:**
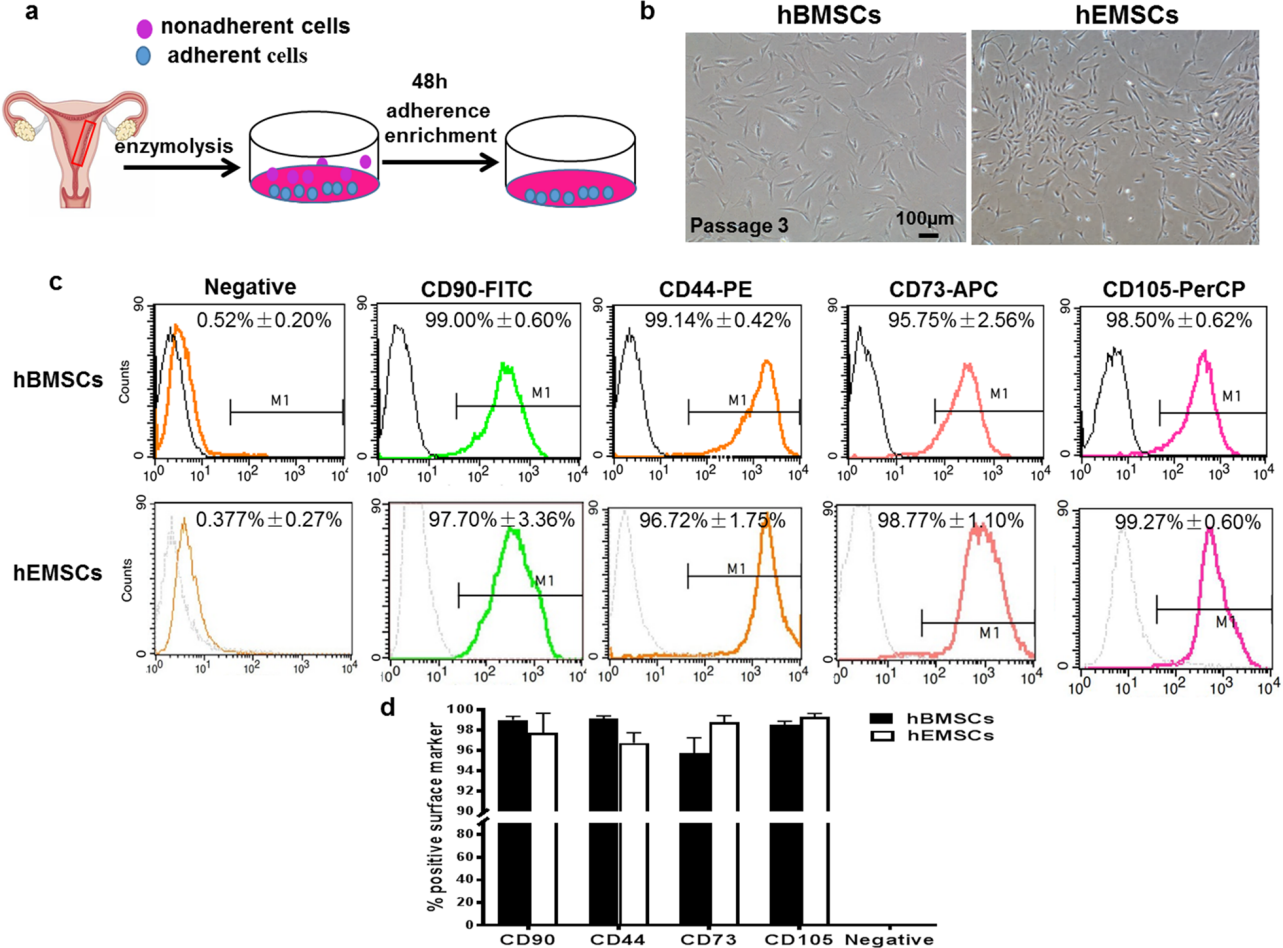
Cultivation and identification of hEMSCs. **a** Schematic illustration of isolating and culturing of human endometrium-derived stem cells (hEMSCs). **b** Representative phase-contrast morphological observation of human bone marrow mesenchymal stem cells (hBMSCs) and hEMSCs cultured up to passage 3 after isolation from premenopausal donors (30–50 years old). **c** Representative histogram plots of cell surface markers from hEMSCs and hBMSCs. **d** Comparison of % positive cells of hEMSCs and hBMSCs. Data are expressed as mean ± SEM. n = 3/group

**Fig. 5:**
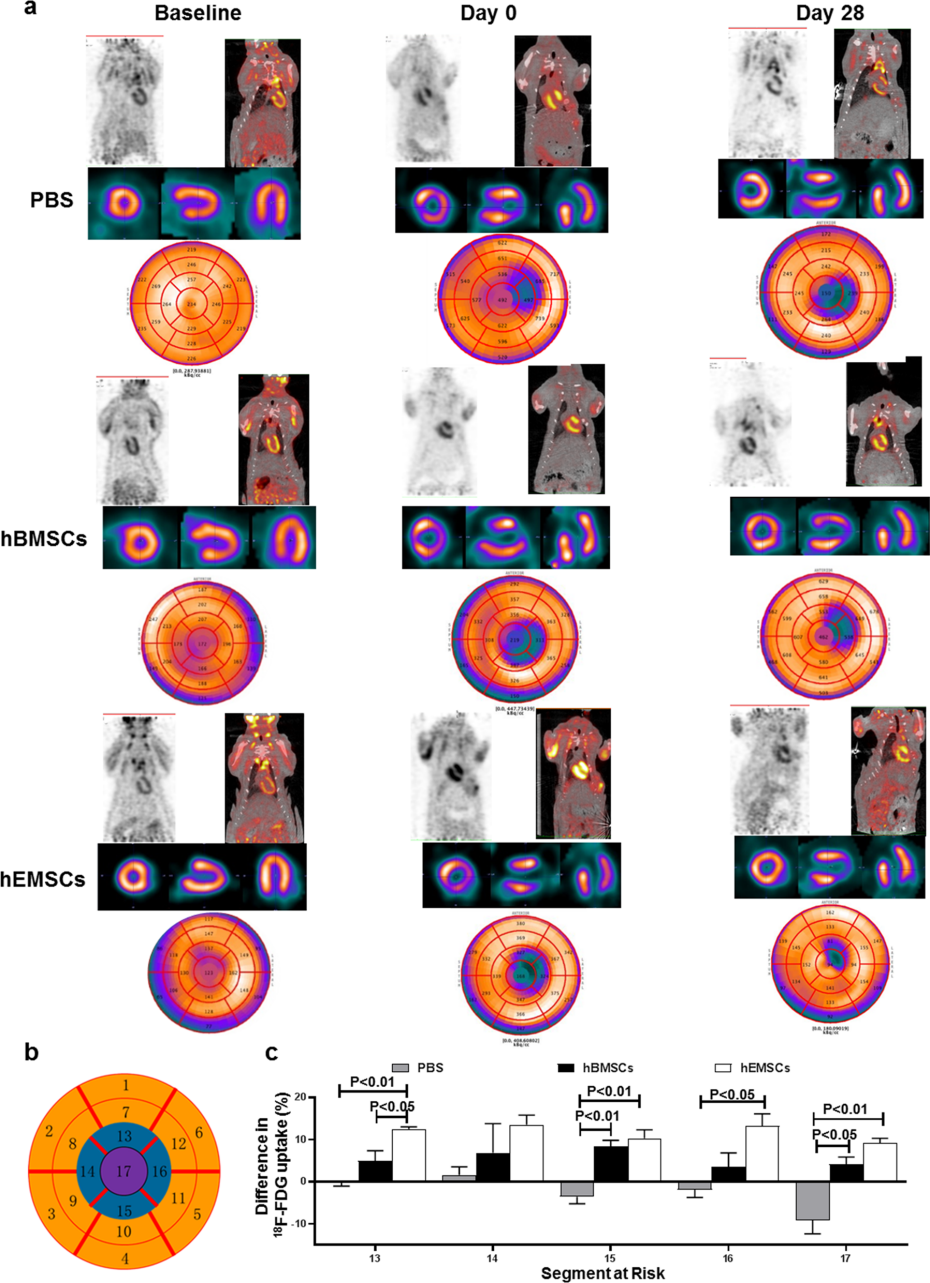
Changes in cardiomyocyte glucose metabolism after MI and hEMSC implantation. Cardiomyocyte glucose metabolism was assessed by ^18^F-FDG uptake using microPET at baseline, and at 0 and 28 days after cell transplantation. **a** Representative transverse, coronal and sagittal ^18^F-FDG uptake images in nude rats that received injection of PBS (PBS control), human bone marrow mesenchymal stem cells (hBMSCs), or human endometrium-derived stem cells (hEMSCs). **b** A pie-shape heart map was used for analysis of cellular metabolism. **c** Quantification of ^18^F-FDG uptake in the apical regions among the three groups. The increase in the uptake of ^18^F-FDG in the apical regions was highest in the hEMSC group. Data are expressed as mean ± SEM. n = 3/group

The corrected 1st image in Fig. 1b, as well as the corrected 2nd image in the first row (Day 0, PBS) in Fig. 5a, is given in this article.

Please note that these corrections do not affect the results and conclusions of our publication and all the authors have agreed on the correction of this negligence. We apologize to the Editor and the readership of the journal for any inconvenience it caused.

