# Supplementary material for: Human endometrium-derived stem cell improves cardiac function after myocardial ischemic injury by enhancing angiogenesis and myocardial metabolism
Source: Stem Cell Res Ther. 2021 Jun 10;12:344. doi: 10.1186/s13287-021-02423-5 (PMC8193887; doi:10.1186/s13287-021-02423-5)
Supplement: Supplementary file 1 — Additional file 1: Fig. S1. Results from RNA-seq and Protein array. a Heatmap from RNA-seq representing expression levels for differentially expressed genes (DEGs) between hBMSCs and hEMSCs. Red: upregulation; Blue: downregulation. Expression intensity was based on R software analysis of gene expression levels. b Twenty categories with significant enrichment were screened for functional enrichment analysis of gene ontology (GO). Yellow line: Number of genes. Bars: Significance level. c Heatmap showing up- and down-regulation for DEGs in hBMSCs and hEMSCs related to angiogenesis. d Venn diagram and protein array analysis between hBMSCs and hEMSCs for DEGs. Black circle in Venn diagram represents DEGs related to angiogenesis between hEMSCs and hBMSCs by RNA-seq, while gray circle represents DEGs identified through protein arrays. The intersection of the two circles represents overlapping DEGs between RNA-seq and protein array for angiogenesis, where five angiogenesis-related factors were found, of which PGF and ANG4 were up-regulated in hBMSCs, while FLT-1, TEK (also known as TIE2), and FGF9 were upregulated in hEMSCs. FGF9: Fibroblast growth factor 9; FLT-1: Fms-like tyrosine kinase 1; PGF: Placental growth factor; ANG4: Angiopoietin 4. Table S1. Formulation of Differentiation Media. Table S2. Real-time qPCR primer sets. [file 13287_2021_2423_MOESM1_ESM.docx]

**Supplementary Information**

for

**Human endometrium-derived stem cell improves cardiac function after myocardial ischemic injury by enhancing angiogenesis and myocardial metabolism**

Xuemei Fan^1, 2^, Sheng He^1,^ ^3^, Huifang Song^1^, Wenjuan Yin^1^, Jie Zhang^1^, Zexu Peng^1^, Kun Yang^1, 2^, Xiaoyan Zhai^1^, Lingxia Zhao^2^, Hui Gong^1^, Yi Ping^4^, Xiangying Jiao^1^, Sanyuan Zhang^3^, Changping Yan^3^, Hongliang Wang^3, 5^, Ren-Ke Li^6*^, Jun Xie^1*^

^1^The Laboratory of Stem Cell Regenerative Medicine Research, Shanxi Key Laboratory of Birth Defect and Cell Regeneration, Key Laboratory of Cell Physiology of Ministry of Education, Shanxi Medical University, Taiyuan, China

^2^Shanxi Bethune Hospital, The Third Hospital of Shanxi Medical University, Taiyuan, China

^3^The First Hospital of Shanxi Medical University, Taiyuan, China

^4^The Second Hospital of Shanxi Medical University, Taiyuan, China

^5^Key Laboratory of Molecular Imaging, Molecular Imaging Precision Medicine Collaborative Innovation Center, Shanxi Medical University, Taiyuan, China

^6^Toronto General Hospital Research Institute, University Health Network, Toronto, Canada

**Supplementary Methods**

**RNA-Sequencing**

RNA-sequencing (RNA-seq) analyses were performed for both hEMSCs and hBMSCs. Purified RNA samples were provided to Wuhan Huada Medical Laboratory Co., LTD., for library construction and sequencing. Briefly, hEMSCs and hBMSCs were inoculated separately into a 10 cm dish at a density of 4x10^5^ cells. The two kinds of cells were collected 48 h after cell inoculation and total RNA was extracted. Quality and quantity checks were performed by means of Fragment Analyzer (Agilent 2100) and real-time quantitative PCR. The library was then constructed according to the manufacturer’s protocols. Sequencing data were obtained using Illumina HiSeqTM 4000. The raw data was analyzed in the following steps: the sequencing data was first filtered with SOAPnuke (v1.5.2) by removing adapter sequences, low-quality base reads (base quality ≤5) and reads with >10% unknown bases. After filtering, the remaining reads were defined as clean reads, and were mapped to the reference genome using HISAT2 (v2.0.4). Clean reads were aligned with the reference encoding gene set using Bowtie 2 (v2.2.5), and gene expression was calculated using RESM (v1.2.12). Based on the gene expression levels in different samples, the heat-map was drawn with Pheatmap. DESeq2 software (v1.4.5) with Q ≤0.05 was used for differential gene expression analysis.

**Protein Array**

HEMSCs and hBMSCs were inoculated separately into a 10 cm dish, at a density of 4x10^5^ cells. Cell culture medium from these two cell types was collected 48 h after cell inoculation. Afterwards, a protein chip containing 440 kinds of human cytokines (GSR-CAA-440) from RayBiotech was used to analyze the expression levels of these soluble cytokines in the medium. Raybiotech software was used to remove the background of the original data, and to screen the differential expression of proteins after the normalization treatment.

**Supplementary Figure**

**
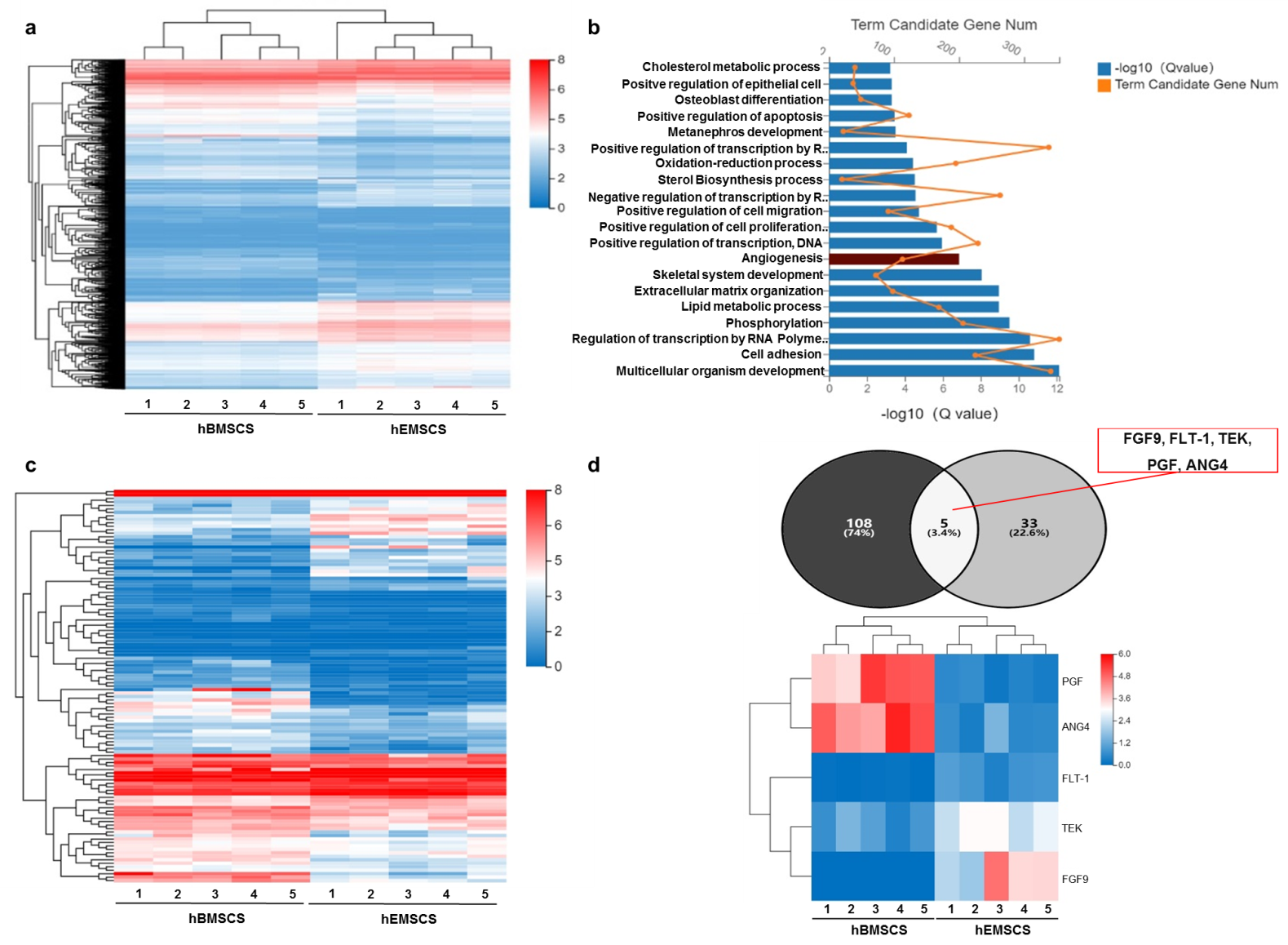
**

**Fig. S1: Results from RNA-seq and Protein array. a** Heatmap from RNA-seq representing expression levels for differentially expressed genes (DEGs) between hBMSCs and hEMSCs. Red: upregulation; Blue: downregulation. Expression intensity was based on R software analysis of gene expression levels. **b** Twenty categories with significant enrichment were screened for functional enrichment analysis of gene ontology (GO). Yellow line: Number of genes. Bars: Significance level. **c** Heatmap showing up- and down-regulation for DEGs in hBMSCs and hEMSCs related to angiogenesis. **d** Venn diagram and protein array analysis between hBMSCs and hEMSCs for DEGs. Black circle in Venn diagram represents DEGs related to angiogenesis between hEMSCs and hBMSCs by RNA-seq, while gray circle represents DEGs identified through protein arrays. The intersection of the two circles represents overlapping DEGs between RNA-seq and protein array for angiogenesis, where five angiogenesis-related factors were found, of which PGF and ANG4 were up-regulated in hBMSCs, while FLT-1, TEK (also known as TIE2), and FGF9 were upregulated in hEMSCs. FGF9: Fibroblast growth factor 9; FLT-1: Fms-like tyrosine kinase 1; PGF: Placental growth factor; ANG4: Angiopoietin 4.

**Supplementary Tables**

**Table S1: Formulation of Differentiation Media**

| **Lineage** | **Differentiation induction media components** | **Staining** |
| --- | --- | --- |
| Osteogenic | Osteogenesis Differentiation Kit (Gibco, Cat#: A1007201) | alizarin red |
| Adipogenic | Isobutyl-methylxanthine (500 µM, Sigma, Cat#: 17018) | oil red |
|  | Rosiglitazone (1 µM, Sigma, Cat#: R2408) |  |
|  | Dexamethasone (1 µM, Sigma, Cat#: D4902) |  |
|  | Novolin R (10 µg/ml, Novo nordisk) |  |

**Table S2: Real-time qPCR primer sets**

| **Gene** | **Forward** | **Reverse** | **species** |
| --- | --- | --- | --- |
| VEGFA | CAAACCTCACCAAAGCCAGC | ACGCGAGTCTGTGTTTTTGC | Rat |
| ANG1 | TGGGCAATGTGCCTACACTT | TGTATCTGGGCCATCTCCGA | Rat |
| ANG2 | CCCCTACATGTCTAACGCCG | CATCACAGCCGTCTGGTTCT | Rat |
| GAPDH | GGCTCATGACCACAGTCCAT | ACATTGGGGGTAGGAACACG | Rat |
| VEGFA | AGGGCAGAATCATCACGAAGT | AGGGTCTCGATTGGATGGCA | Human |
| ANG1 | GCAGCAGATAGGGTAGAGGA | TGTTTCCTCTCTGTGTGACCG | Human |
| ANG2 | CTCGAATACGATGACTCGGTG | TCATTAGCCACTGAGTGTTGTTT | Human |
| GAPDH | GGAGCGAGATCCCTCCAAAAT | GGCTGTTGTCATACTTCTCATGG | Human |

ANG1: angiopoietin 1; ANG2: angiopoietin 2; GAPDH: glyceraldehyde 3-phosphate dehydrogenase
